# Electromagnetic characterization of mirror symmetric resonator based metamaterial and frequency tuning: a dielectric based multilayer approach

**DOI:** 10.1038/s41598-022-16443-5

**Published:** 2022-07-21

**Authors:** Md. Moniruzzaman, Mohammad Tariqul Islam, Md Samsuzzaman, Abdullah G. Alharbi, Mohamed S. Soliman, Norbahiah Misran, Md. Shabiul Islam

**Affiliations:** 1 Department of Electrical, Electronic and Systems Engineering, Faculty of Engineering and Built Environment, Universiti Kebangsaan Malaysia, Bandar Baru Bangi, Malaysia; 2 Department of Computer and Communication Engineering, Faculty of Computer Science and Engineering, Patuakhali Science and Technology University, Patuakhali, Bangladesh; 3 Department of Electrical Engineering, Faculty of Engineering, Jouf University, Sakaka, 42421 Saudi Arabia; 4 Department of Electrical Engineering, College of Engineering, Taif University, P.O. Box 11099, Taif, 21944 Saudi Arabia; 5 Department of Electrical Engineering, Faculty of Energy Engineering, Aswan University, Aswan, 81528 Egypt; 6 Faculty of Engineering, Multimedia University, 63100 Cyberjaya, Selangor Malaysia

**Keywords:** Metamaterials, Electrical and electronic engineering

## Abstract

This article presents a novel metamaterial (MTM) with a mirror symmetric resonator that exhibits multiple resonances of transmission coefficient covering the L, S, C, and X bands. The resonating patch is constructed on a low-loss Rogers (RT5880) substrate with a dimension of 20 × 20 × 1.575 mm^3^. It consists of four equal quartiles with each quartile made with interconnected split-ring resonators; the quartiles are in mirror symmetry with each other. The proposed MTM exhibits resonances at 1.98 GHz, 3.67 GHz, 4.74 GHz, 8.38 GHz, and 10.8 GHz, and electromagnetic characterization is performed through studies of permittivity, permeability, refractive index, and impedances. Power analysis is also performed, and the effect of polarized incident waves is studied. An electromagnetic characterization study reveals that the proposed MTM shows negative permittivity with near-zero permeability and refractive index. It also reveals very little power consumption in the vicinity of the resonances. The dielectric-based frequency tuning is studied by using different dielectric layers over the patch that provides good frequency tuning; this method provides flexibility for adjusting the resonance frequencies in accordance with the application demand. The measured result of the proposed unit cell with the dielectric layer is extracted using a vector network analyzer, and the results exhibit good similarity with the simulated ones. The above-mentioned properties, along with a good effective medium ratio (EMR) of 7.57 indicate that this MTM is suitable for frequency selective applications in microwave devices such as antenna performance improvement and sensing.

## Introduction

Metamaterial that reveals several amazing properties finds great attraction among researchers for its various applications in the microwave, near-infrared, and visible spectrum^1,2^. Several interesting phenomena, including superlensing^3^ and invisible cloaking^4^, are obtained by tailoring the refractive indexing exposed by a metamaterial. However, its epsilon-negative property leads to its applicability for boosting the gain and size reduction of the antenna^5,6^ for wearable microwave devices^7^ and filter design^8^. Mu-negative metamaterials are employed for isolation purposes of MIMO antennas^9^, beam focusing of the wideband antenna^10^, and reduction of leaking of electromagnetic waves in wireless power transfer^11^. Moreover, microwave sensors^12^, absorbers^13^, etc. are other utilizations of metamaterial for various sensing and detection applications. Thus, research on metamaterial embraces an ever-increasing demand that explores different material and structural design, as well as various techniques of attaining desired properties targeting applications.

In recent works, Alibakhshikenari et al. presented a metamaterial periodic structure that is utilized as decoupling media for isolating purposes in MIMO antenna systems^14^. The MTM with photonic band gap is applied in this system that has a cross-shaped microstrip structure. An MTM-based sensor is introduced that consists of triple hexagonal-shaped split-ring resonators at the front and back of the substrate^15^. This MTM is used as a sensor for fuel adulteration using the concept of frequency shifting due to the variation of dielectric properties. Yang et al. presented a metamaterial-based polarization converter that consists of a spiral-shaped split-ring resonator. The integration of two orthogonal gratings helps to improve conversion efficiency to 90% by rotating linear polarization waves in an orthogonal direction^16^. Lie et al. demonstrated a metamaterial with an asymmetric structure that exhibits toroidal dipole resonance with the potential for applications in meta-devices covering microwave regimes^17^. By contrast, Wang et al. presented a metamaterial with double split-ring resonators that exhibits fano resonances in terahertz frequencies^18^. By using position asymmetry and gap asymmetry, fano resonances are tuned in this design, and the Q factor with the figure of merit is optimized. A meander line resonator based metamaterial is presented in ^19^ in which resonance frequencies can be tuned by using switches placed at split gaps of three rings. Moreover, Xu et al. presented a tunable metamaterial that operates in infrared regions; it acts as an emitter with high efficiency that is realized with the integration of a micro heater, an electromechanical system, and a metamaterial. In this system, the metamaterial helps to narrow down the radiation spectrum emitted from the micro heater and eventually makes wavelength selective with narrow beam emission^20^. A perfect metamaterial absorber that exhibits more than 99% absorption within the frequency range 6.6–8.9 THz was presented by Zhang et al.. Dynamic tuning provides flexibility for controlling absorption in the terahertz range, and this VO_2_ base metamaterial absorber is suitable for modulation and filtering^21^. Several metamaterial reflectors are presented in different works of literature targeting various applications, design patterns, and characteristics studies^22–24^. A single negative metamaterial was presented by Azeez that consists of concentric rings and a cross-line resonator on an FR4 substrate^25^. This symmetrical structure operates in the Ku and K bands. Another MTM with a symmetrical resonator is offered in ^26^ and comprises an FR4 substrate with cross-coupled split-ring resonator; the response of this MTM covers the C, X, and Ku bands. An MTM with a resonator having complementary rings was discussed by Goa et al.; this exhibits flexibility with a frequency selectivity property^27^. The frequency selectivity is performed with a ferrite material at the opposite side of the resonator having a thickness of 0.3 mm. A tunable metamaterial that contains a symmetric resonator was presented in recent work; its frequency can be tuned by adjusting tuning metallic stubs positioned along two perpendicular axes passing through center of the resonator^28^. However, the effect of the polarized signal is not studied in this manuscript. For chemical detection, Abdulkarim et al. presented a G-shaped resonator-based MTM that operates within the frequency range of 8–12 GHz; frequency shifting occurs due to dielectric parameter change for different chemical samples^29^. A symmetrical resonator-based MTM was presented by Ramachandran et al. that claims polarization-independent characteristics of a very simple MTM constructed with several circular rings^30^. However, this MTM exhibits only dual-band resonances at 8.667 GHz and 13.8 GHz with a low EMR value of 4.3. On the other hand, a symmetrical metamaterial is presented by Moniruzzaman et al. that can be applied for antenna performance enhancement^31^. Abdul Karim et al. presented a broadband metamaterial absorber that incorporates water between two SiO_2_ layers; this absorber shows broadband absorption characteristics extending from 10.4 to 30 GHz^32^. On the other hand, a thin substrate material of zinc selenide with a thickness of 600 nm exhibits 98.4% absorption at 22.46 THz and 99.28% absorption at 28.95 THz^33^. A Metamaterial has been presented in recent literature which can be applicable for antenna gain enhancement. Inductively tuning mechanism is used in this design to shift the resonance towards the lower frequencies. Moreover, with copper backplane this MTM exhibits good absorption properties. But, due to lack of complete symmetry, the MTM array shows a frequency deviation in response compared with the unit cell^34^.

In this manuscript, a novel symmetric metamaterial that offers an MTM exhibiting multiple resonances of S_21_ covering the L, S, C, and X bands is presented. The electromagnetic properties of this metamaterial are analyzed, and multilayer dielectric-based tuning is studied in simulation and experimentation. The major features of the proposed MTM can be summarized as follows: (i) the MTM is constructed with a combination of four equal quartiles; (ii) each quartile is a mirror image of the other quartile placed in the same row or column; (iii) this approach makes the total unit cell mirror symmetrical about the two perpendicular axes; (iv) due to the mirror symmetrical structure of the design, harmonics are eliminated, and an array of the unit cells shows a response similar to that of the unit cell; (v) moreover, frequency-tuning of the MTM using a dielectric layer over the resonating patch provides further flexibility for adjusting the frequency of resonances for particular applications; and (vi) additionally, this design provides moderate EMR value with negative permittivity/epsilon negative (ENG) and near zero index (NZI) properties with multi-frequency responses covering the L, S, C and X bands. Thus, the MTM has the potential for applications in various microwave devices for wireless communication and non-metallic object-sensing purposes. The rest of the article is arranged as follows. The design and simulation of the offered MTM are described in section two. In section three, frequency-tuning using a dielectric layer is studied through simulation and theoretical studies. Result analysis and discussion are provided in section four, which includes extraction and analysis of the effective parameters, power, and analysis of measured results for the MTM, and frequency-tuning using dielectric layers. Finally, a comparison of this MTM is made with those of several recently published works. In the subsequent section, a conclusion includes the key outcomes of this study.

## Design methodology of the proposed MTM unit cell

**Figure 1:**
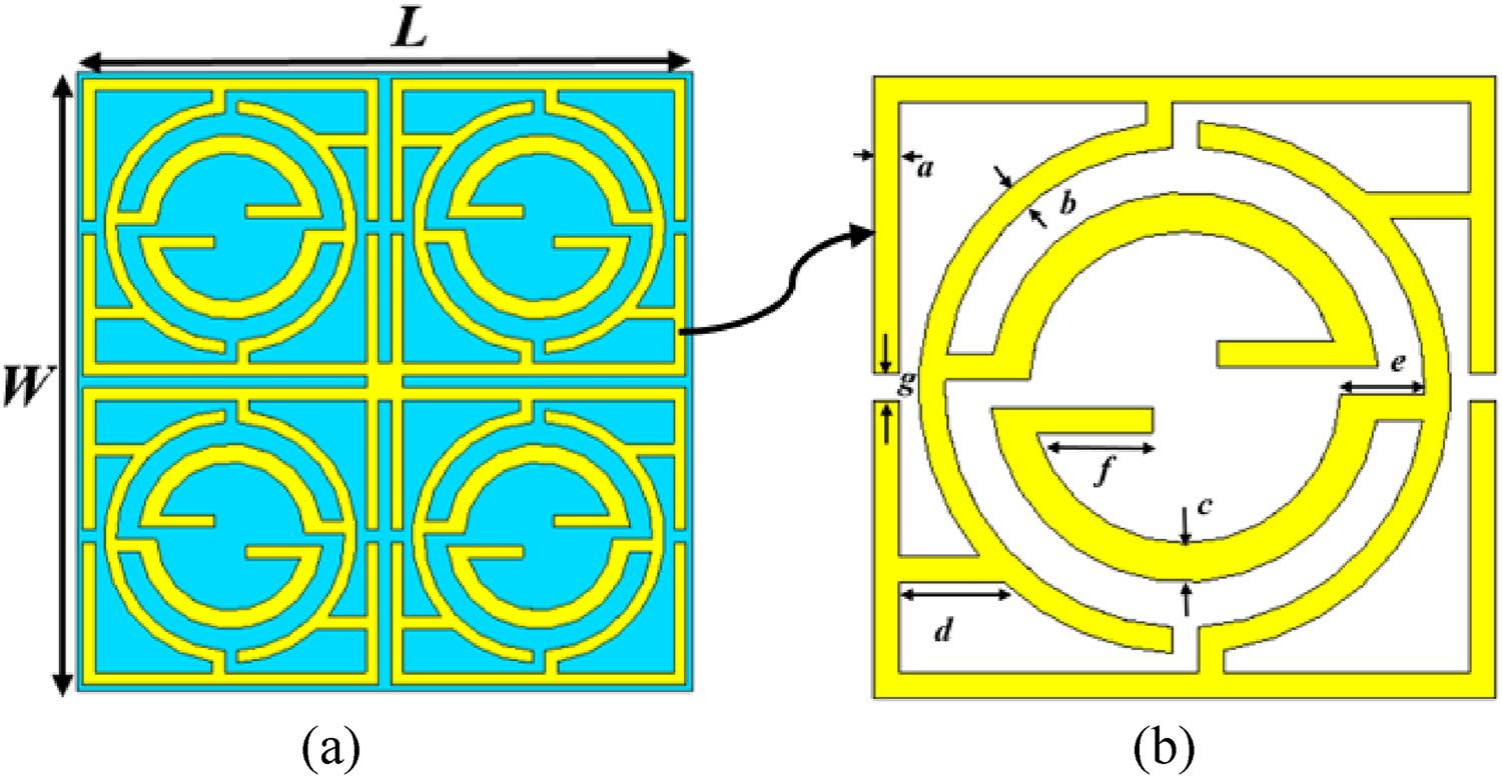
Front view of the proposed MTM unit cell: (**a**) total patch layout, (**b**) enlarged view of a segment of the patch (CST microwave studio suite 2019).

**Figure 2:**
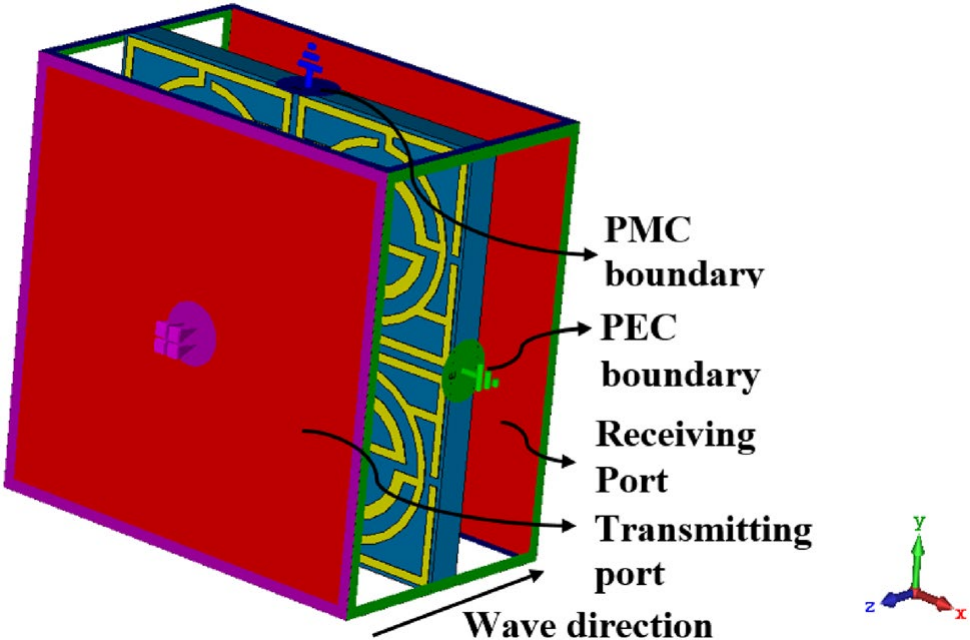
Simulation arrangement of the MTM unit cell (CST microwave studio suite 2019).

The proposed MTM unit cell is constructed on a Rogers substrate (RT5880) with the dimensions 20 mm × 20 mm × 1.575 mm, as expressed in Fig. 1a. The resonator patch is constructed with copper with a thickness of 0.035 mm at one side of the substrate; the other side contains no metallic elements. The resonating patch can be subdivided into four equal segments that are connected at the center by a square metal strip. The placement of the segments on the substrate is such that each one is mirror symmetrical to the adjacent segments, as depicted in Fig. 1a. Each segment of the resonator contains three interconnected split rings and interconnections as expressed in Fig. 1b. Moreover, split gaps and other metal strips are placed in such a way that the segment itself is inverse symmetric in two perpendicular axes extended through the center of each segment. The total structure is chosen so that when a group of unit cells is arranged in a regular array, and when this is placed in the excited electromagnetic field, the cross-coupling effect between the cells is eliminated due to the mirror symmetric structure, and the array acts like a unit cell and exhibits a similar performance. The dimensions of the rings and split gaps are adjusted in simulation for optimization of appropriate MTM structure to attain the preferred response of the proposed MTM. For this optimization, several numerical simulations are performed by changing the dimension, position, split gaps of the rings, interconnecting metallic stubs position, inter ring distance, and thus, the appropriate structure is selected that provides the preferred response. In Table 1, the optimized dimensions of different segments of the MTM are included. The 3D structural simulation of the MTM cell is performed using CST microwave studio of 2019 version, and simulation setup is presented in Fig. 2. In the simulation process, the MTM is positioned in middle of two ports as illustrated in Fig. 2, and a perfect electric boundary condition is applied in the X direction, whereas in the Y direction, a magnetic boundary is applied. The transverse electromagnetic (TEM) wave is propagated in the Z direction and falls upon the resonator perpendicularly. The incident electromagnetic waves excite the resonator patch, and electromagnetic interaction occurs within the MTM unit cell that creates resonance in the transmitted and reflected waves. The simulation is performed within the frequency ranges 1–12 GHz.

**Table 1:** Dimension of the various segments of the MTM.

Parameter	Dimension (mm)	Parameter	Dimension (mm)	Parameter	Dimension (mm)
*W*	*20*	*L*	20	*a*	0.44
*b*	0.4	*c*	0.6	*d*	1.6
*d*	1.3	*f*	1.8	*g*	0.4

### Evolution of the MTM unit cell

**Figure 3:**
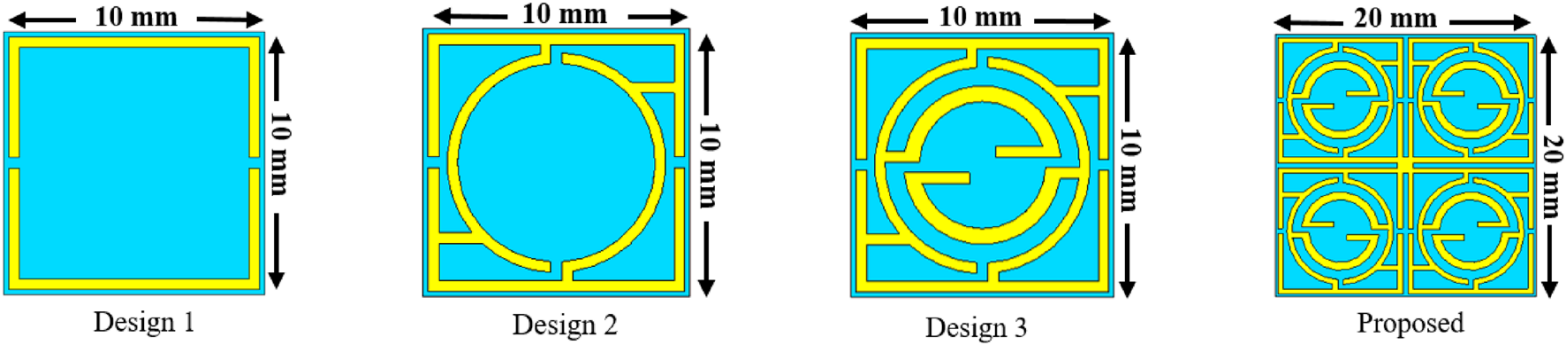
Design steps towards the proposed MTM unit cell (CST microwave studio suite 2019).

**Figure 4:**
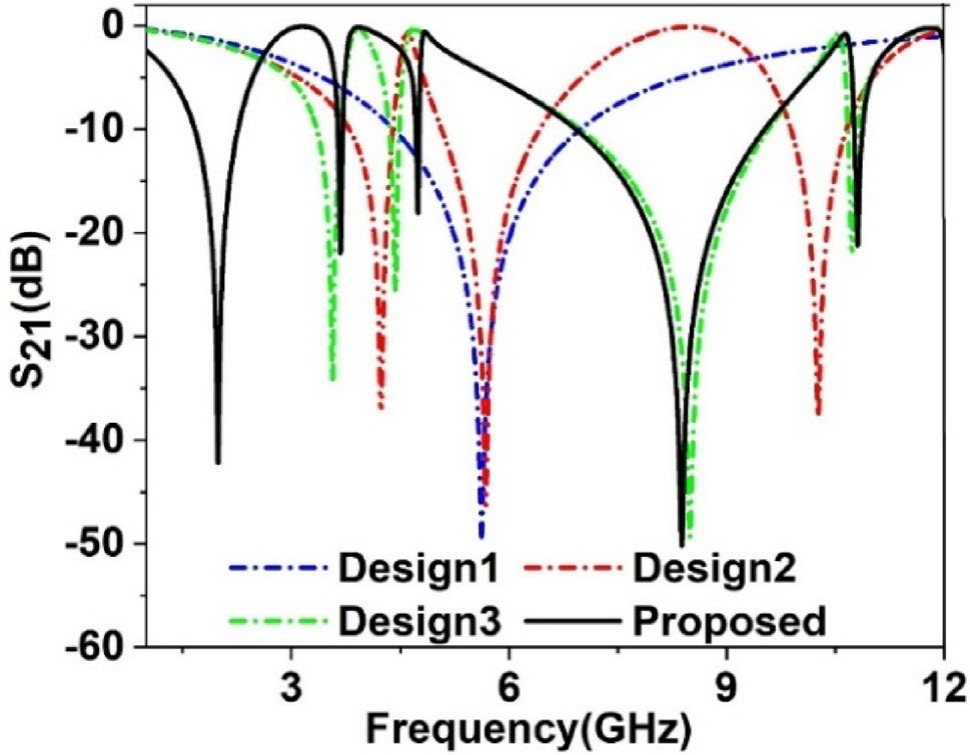
Transmission coefficient for all design steps towards the finalized MTM unit.

The design-evolution steps of the proposed MTM are shown in Fig. 3. The design is initiated primarily on a substrate of 10 × 10 mm^2^ with a square-shaped resonator consisting of two split gaps at the vertical arms, as illustrated by Design 1 in Fig. 3. This split-ring resonator provides a wideband resonance of transmission coefficient (S_21_) at 5.6 GHz, as shown in Fig. 4. Now, another circular-shaped split ring is added within this outer square-shaped ring that contains split gaps along the vertical axis and is interconnected with the outer one at four sides in such a way that an inverse symmetry structure is obtained, as expressed by Design 2 in Fig. 3. These interconnected split-ring resonators provide three resonances taking place at 4.2 GHz, 5.7 GHz, and 10.3 GHz, as depicted in Fig. 4. The effect of this additional interconnected ring is obvious, with a shifting of resonance frequency and bandwidth of the initial resonance. Moreover, it causes two additional resonances occurring at 4.2 GHz and 10.3 GHz. Design 3 of Fig. 3 includes another circular ring that contains two split gaps; this ring is connected to the closest ring near the split gaps like the inverse symmetric. Moreover, two extended metal strips are added at the site of the split gaps that are produced toward the center of the ring. This insertion causes four resonances at 3.57 GHz, 4.43 GHz, 8.5 GHz, and 10.72 GHz covering the S, C, and X bands, respectively. In the final design step, four units of Design 3 are placed in a 2 × 2 array to form the mirror-symmetric pattern, and these four units are interconnected at the center of the base using a small piece of the rectangular copper strip as displayed in Fig. 3. Thus, the proposed MTM unit cell that is formed is not only mirror symmetric but also exhibits rotating symmetry, which helps to eliminate the harmonics due to the coupling effect when an array is formed using unit cells. Thus, this symmetric pattern of the proposed MTM helps to create a similar response between array and unit cell, and the metamaterial acts as a meta-atom. The S_21_ response of this final structure is exhibited at 1.99 GHz, 3.67 GHz, 8.38 GHz, 4.74 GHz, and 10.8 GHz covering L, C, S, and X bands that are illustrated in Fig. 4. The performances exhibited by the MTM at different evaluation steps have been summarized in Table 2. In our proposed MTM, all the rings are interconnected to each other. Thus the inductance associated with each ring is coupled to each other using the interconnected metallic stubs. For this reason, the changing effect of the geometrical size of any ring causes variations of all resonances. For brevity, the simulated results of various dimensions of the rings are not included here. As the rings are not separated from each other thus, any resonance frequency cannot be independently tuned by the change in the geometrical size of a particular section of the total resonator.

**Table 2:** Performance comparison for different design-evolution steps toward the proposed unit cell.

Design steps	Frequency of resonance	Bandwidth (GHz)	Covering bands	EMR
Design 1	5.6	2.54	C	5.4
Design 2	4.2, 5.7, 10.3	0.62,1.27, 0.75	C, X	7.14
Design 3	3.6, 4.4, 8.5, 10.7	0.34, 0.15, 2.6, 0.14	C, X	8.3
Proposed MTM	1.98, 3.67, 4.74, 8.38, 10.8	0.64, 0.07, 0.05, 2.64, 0.11	L, S, C, X	7.5

### Equivalent circuit modeling of the MTM

**Figure 5:**
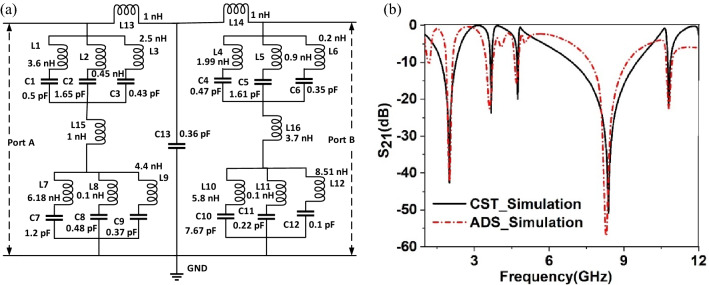
(**a**) MTM’s equivalent circuit, (**b**) S_21_ plot of circuit simulation in ADS compared with 3D structural simulation in CST.

To understand the exotic behavior under incident electromagnetic waves, an equivalent circuit has been modeled in which the resonator of the MTM can be replaced with the equivalent LC circuit. In our proposed model, each quartile of the unit cell consists of three split rings interconnected with each other. These three interconnected split rings are resembled by three parallel-connected LC circuits in which metallic rings exhibit an inductive property, whereas split gaps in the ring exhibit capacitances. When an electromagnetic wave interacts with the resonator, resonance occurs due to these inductances and capacitances. Thus, we obtain resonances whose frequency depends upon the inductance and capacitance values of the resonator. By controlling rings’ length as well as width, and inter ring distances, the inductance property can be modified, whereas capacitance values can be modified by changing the split-gap distances. Since three parallel-connected LC branches are presented by each quartile, four such branches are used in our equivalent circuit, and each group represents each quartile. In the equivalent circuit, interconnecting copper strip that connects four quartiles is replaced by inductances L13, L14, L15, and L16. Capacitor C13 represents the lumped parasitic capacitance that exists in the MTM cell due to the separation of the rings. The equivalent circuit is depicted in Fig. 5a, in which two ports are connected at the two ends of the circuit. After modeling the circuit, it was designed and simulated using Advanced Design System (ADS) software and verified by comparing performance with CST simulation. In ADS simulation, ports are terminated with 50-Ohm impedance. The initial values of all discrete components are selected as 1 nH for each inductor and 1 pF for each capacitor. Next, the component values are tuned through the tuning module of the ADS, and component values are finalized by comparing the S_21_ response of the equivalent circuit in ADS with the same response obtained in CST. Figure 5b shows the plots of the S_21_ response for both equivalent circuit and 3D design in CST. As depicted in Fig. 5b, both S_21_ exhibit close similarity; thus, it is determined that the equivalent circuit represents the circuit model of the MTM unit cell.

## Frequency tuning using the dielectric layer

In a metamaterial, when an incident wave is imposed, electromagnetic interaction occurs between the metamaterial and incident wave. Due to the inductive property of the copper stripe and the capacitance due to the split gaps, and the coplanar capacitance due to the gap between the metal rings, the whole structure acts as an LC resonance circuit. For this reason, electromagnetic resonance occurs in the metamaterial, and resonances happen with resonance frequencies depending on the values of the inductance and capacitances of the total structure and can be defined as:

1 $$f=\frac{1}{2\pi }\sqrt{\frac{1}{LC}}$$

where *f*, *L,* and *C* express the frequency of resonance, inductance, and capacitance, respectively. An increasing effective permittivity causes the capacitance to increase; thus, the resonance frequency of the SRR decreases with the increasing effective permittivity^35^. If a dielectric material is placed over the structure, the incident field interacts with it, and due to this interaction, the overall characteristics of the MTM structure change with a modification of the effective permittivity^36^. The thickness of the dielectric layer over the MTM also affects the effective permittivity, and an increased thickness results in increasing the effective permittivity^35^.

**Figure 6:**
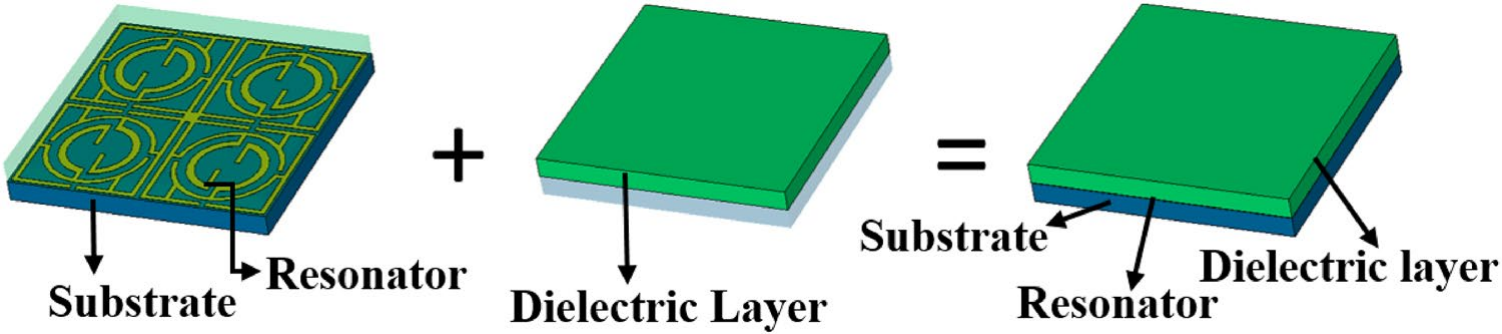
Proposed MTM unit cell with a dielectric layer over the resonator patch (CST microwave studio suite 2019).

**Figure 7:**
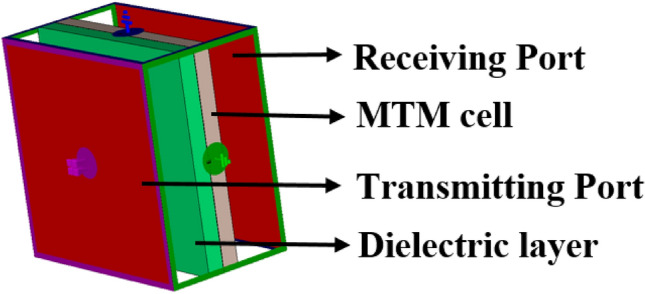
Simulation arrangement for dielectric layer with MTM (CST microwave studio suite 2019).

**Figure 8:**
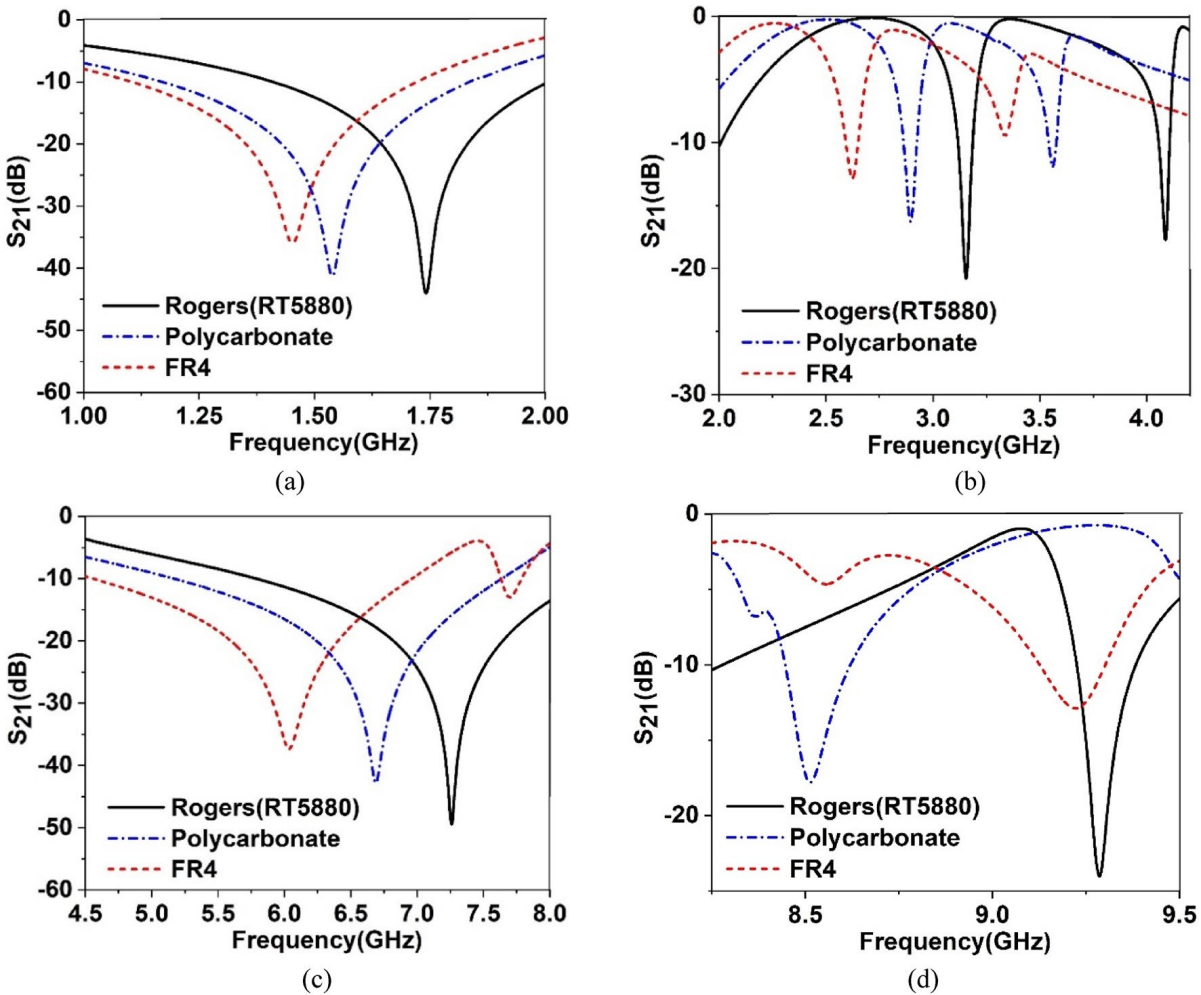
S_21_ response of the proposed MTM with Rogers(RT5880), Polycarbonate and FR4 dielecrtic layer over resonator within: (**a**) 1–2 GHz, (**b**) 2–4 GHz, (**c**) 4.5–8 GHz, (**d**) 8.2–9.5 GHz.

Therefore, to change the overall capacitance associated with the metamaterial, a separate dielectric material can be used over the resonator patch so that the resonator patch is sandwiched between Rogers (RT5880) substrate and the newly imposed dielectric material, as shown in Fig. 6. The effect of imposing a separate dielectric layer over the resonator patch is studied by numerical simulation using three available materials: Rogers (RT5880), FR4, and polycarbonate with thicknesses of 1.57 mm, 1.6 mm, and 1.5 mm, respectively. The values of permittivities are 2, 4.4, and 2.9 for Rogers (RT5880), FR4, and polycarbonate, respectively, with the loss tangent values of 0.02, 0.0021, and 0.01, respectively. In a simulation setup as shown in Fig. 7, the ports are arranged so that an incident electromagnetic wave is passed through the air, then imposed on the implied dielectric layer on the way to the unit cell. When any of the above-mentioned materials are used as a layer over the resonator, the incident electromotive wave interacts with this nonmetallic layer and then falls on the resonating patch and back substrate. Thus, a cumulative effect takes place that ultimately exhibits an impact on the transmitting as well as reflecting wave, as discussed earlier. A study of the effect of this dielectric layer on the transmission coefficient is performed, and graphs of S_21_ are plotted in Fig. 8a–d for each of the three nonmetallic layers. By observing the S_21_ pattern presented in Fig. 8a–d, it can be seen that each dielectric material causes changes in resonance frequencies of the proposed MTM. Moreover, −10 dB bandwidth and magnitude of S_21_ are also modified as an effect of these dielectric materials. A summary of the S_21_ response is presented in Table 3 and can help make possible a closer comparison between the outputs for different nonmetallic layers along with the output of the proposed MTM structure. Table 3 shows that when the dielectric medium is used as an extra layer over the resonating patch, the resonance frequency shifts toward the lower values, depending on the material properties, with a maximum shift in the case of FR4 materials and a minimum for Rogers (RT5880), indicating that resonance frequency depends mainly on the permittivity of the dielectric material. The higher the permittivity is, the higher the shift in the resonance frequency toward the lower frequency band is. However, another factor is also observed from the plots presented in Fig. 8a–d, which is that the negative magnitude of the transmitted signal is high for the Rogers (RT5880) and low for the FR4 substrate. Of these three materials, the loss tangent value is higher for the FR4 substrate which substantially affects the magnitude at the resonance of S_21_. It is worth noting that, for the resonances at the high frequencies, the maximum shift in resonance frequencies is noticed, whereas the S_21_ responses at the low frequency undergo a minimum change in resonance frequencies. In addition, the EMR value improves significantly due to the application of the dielectric layer. Thus, frequency tuning using a dielectric layer provides the flexibility to adjust the resonance frequency of the proposed MTM according to the application requirement. Moreover, covering material can also be detected through this frequency shifting that can be applied for sensing various objects^35^.

**Table 3:** Comparison of the effect of different dielectric layers over the resonator.

Material of the dielectric layer	Resonance frequency (GHz)	Bandwidth (GHz)	Resonance shift (GHz)	Covering bands	EMR
Roger(RT5880)	1.74, 3.16, 4.09, 7.26, 9.3	0.6, 0.07, 0.1, 2.48, 0.15	0.26, 0.51, 0.65, 1.12, 1.5	L,S,C,X	8.6
FR4	1.46, 2.6, 6.1, 7.7, 9.2	0.62, 0.06, 2.38, 0.15, 0.17	0.54, 1.07, 2.28, 3.1	L,S,C,X	10.3
Polycarbonate	1.54, 2.9, 3.56, 6.7, 8.52	0.69, 0.07, 0.18, 2.48, 0.17	0.46, 0.77, 1.18, 1.64, 2.28	L,S,C,X	9.7

## Result analysis and discussion

The performance of the proposed MTM is further studied through the analysis of its effective parameters in terms of permittivity, permeability, refractive index, and normalized impedance, along with the surface current, magnetic, and electric field analysis. Moreover, the power associated with this MTM is analyzed. The effect of the polarized incident wave on the MTM is also observed along with the polarization conversion ratio (PCR). The experimental result is included for the proposed MTM and different tuning nonmetallic layers in this section. The comparison with other states of the arts is included here as well.

### Effective parameters of the proposed MTM

**Figure 9:**
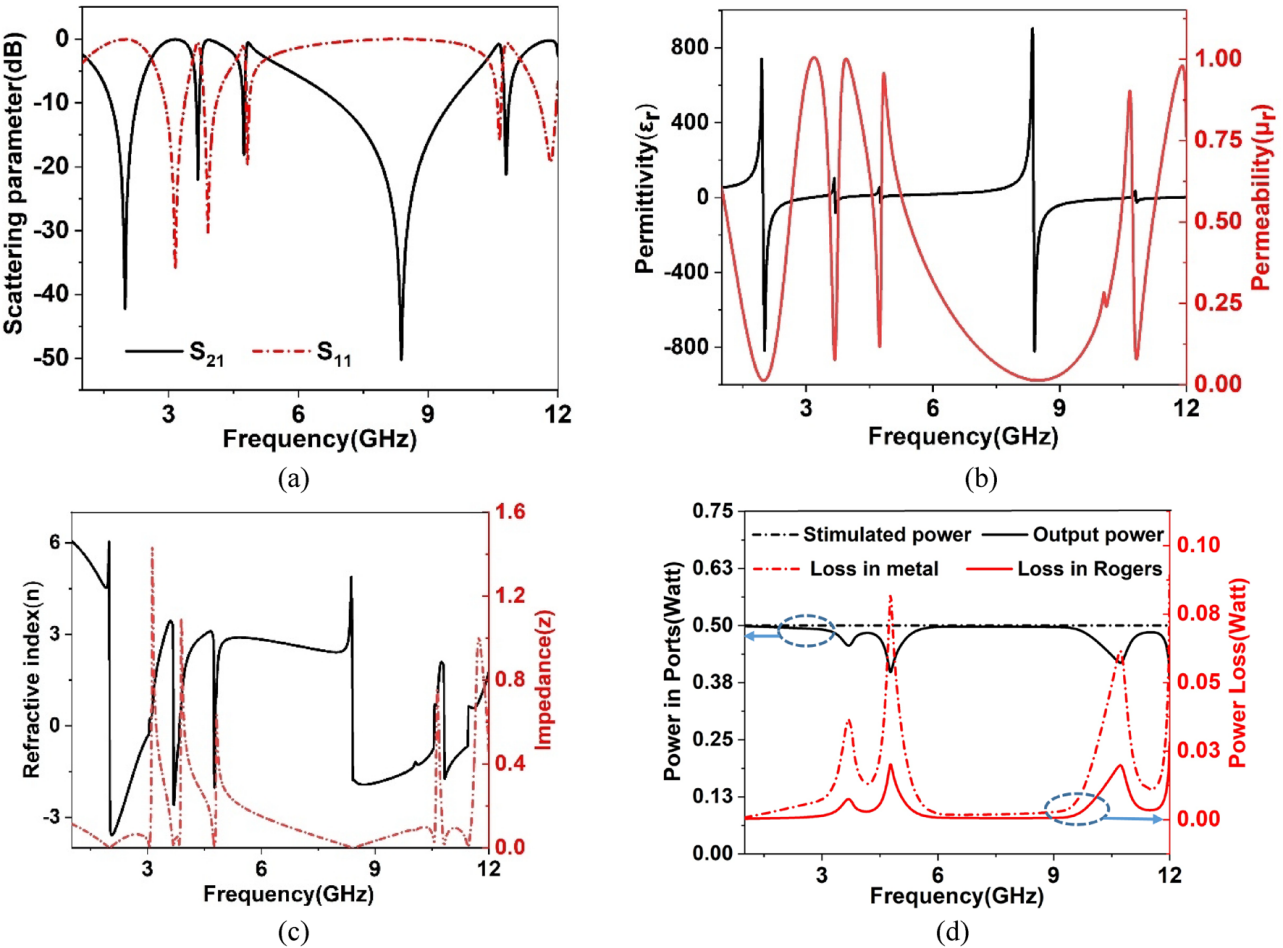
(**a**) scattering parameters(S_21_ and S_11_), (**b**) permittivity and permeability, (**c**) refractive index and impedance (**d**) power.

The proposed MTM interacts with the incident electromagnetic wave, and resonance occurs in the reflected and transmitted signal. The resonance pattern of these signals is expressed in Fig. 9a, which illustrates S_21_ resonances at 1.98 GHz, 3.67 GHz, 4.74 GHz, 8.38 GHz, and 10.8 GHz with the bandwidth extended from 1.66 to 2.24 GHz, 3.64–3.7 GHz, 4.7–4.76 GHz, 6.9–9.56 GHz, and 10.76–10.87 GHz, respectively. Additionally, as depicted in the same figure, the reflection coefficient shows resonance at 3.16 GHz, 3.9 GHz, 4.8 GHz, 10.7 GHz, and 11.8 GHz. It is observed that the proposed MTM exhibits a broadband response of the transmitted signal within 6–8 GHz. A wideband response is also observed within 1–3 GHz that covers the L and C bands. Other resonances are of narrowband and, altogether, the resonances cover the L, S, C, and X bands, respectively. The electromagnetic properties of the MTM have been extracted using a CST post-processing template that uses the transmission (S_21_) and reflection (S_11_) coefficients to calculate permittivity, permeability, and refractive index with the help of a robust retrieval method^37^ where scattering parameters are concerned by:

2 $${S}_{11}=\frac{{R}_{01}\left(1-{e}^{i2n{k}_{0}d}\right)}{1-{{R}_{01}}^{2}{e}^{i2n{k}_{0}d}}$$

3 $${S}_{21}=\frac{\left(1-{{R}_{01}}^{2}\right){e}^{in{k}_{0}d}}{1-{{R}_{01}}^{2}{e}^{i2n{k}_{0}d}}$$

4 $$\mathrm{where}, {R}_{01}=z-1/z+1$$

5 $${e}^{in{k}_{0}d}=X\pm i\sqrt{1-{X}^{2}}$$

6 $$X=1/{2S}_{21}\left(1-{S}_{11}^{2}+{S}_{21}^{2}\right)$$

7 $$\mathrm{Normalized }\, \mathrm{impedance}, z=\pm \sqrt{\frac{{\left(1+{S}_{11}\right)}^{2}-{{S}_{21}}^{2}}{{\left(1-{S}_{11}\right)}^{2}-{{S}_{21}}^{2}}}$$

8 $$\mathrm{refractive\, index}, n=\frac{1}{{k}_{0}d}{\left\{\left[{\left[ln\left({e}^{in{k}_{0}d}\right)\right]}^{{{\prime\prime}}}+2m\pi \right]-i{\left[ln\left({e}^{in{k}_{0}d}\right)\right]}{^{\prime}}\right\}}$$

where (.)’ is the real part, (.)’’ is the imaginary part, and m is an integer related to the real part of the refractive index, ${k}_{0}$ is the wave number in free space- for incident wave, and $d$ is the substrate thickness. Two other effective parameters, permittivity, and permeability can be calculated using the equations:

9 $$\mathrm{permeability}, \mu =nz$$

10 $$\mathrm{permittivity}, \varepsilon =n/z$$

The actual values of the obtained results are depicted in Fig. 9b,c. The permittivity value presented in Fig. 9b shows a sharp resonance at 1.98 GHz with a negative value extended up to 3.2 GHz, whereas a resonance peak of 100 is obtained at 3.67 GHz. The other resonances of the permittivity are obtained at 4.73 GHz, 8.36 GHz, 10.8 GHz, and a group of negative permittivity bands extended in the frequency ranges 3.67–3.89 GHz, 4.74–4.8 GHz, 8.38–9.4 GHz, and 10.8–11.2 GHz. From the permeability graph represented in Fig. 9b, near-zero permeability is attained within the frequency ranges 1.54–2.36 GHz, 3.63–3.71 GHz, 4.7–4.76 GHz, 6.55–9.92 GHz, and 10.77–10.9 GHz. The refractive index plot illustrated in Fig. 9c indicates that it transits from positive to negative values at the frequencies of resonances of S_21_. Thus, near-zero refractive index is obtained near the frequency of resonances, and MTM exhibits a near-zero refractive index property. Moreover, the real part of normalized impedance presented in Fig. 9c indicates that in the vicinity of the resonances, a small amount of power was dissipated in these frequency regions. The power associated with the metamaterial is studied using the plot presented in Fig. 9d. The power transmitted by port 1 is about 0.5 Watt and most of the power is received by port 2 with a negligible amount of loss in the metamaterial. Dips in output power are observed at 3.7 GHz, 4.7 GHz, and 10.7 GHz. These depressions in the output power are due to loss peaks for dielectric and metals. Dielectric losses associated with these frequencies are 1.8%, 1.6%, and 5.4%, which are insignificant compared to the total input power. However, metallic losses are about 9.8%, 6%, and 17% at these frequencies, indicating a relatively high loss. Nevertheless, these loss maxima or output power minima take place near stop-bands; thus, the proposed MTM acts as low-loss media in our target band of frequencies.

### Surface current, electric, and magnetic field analysis

**Figure 10:**
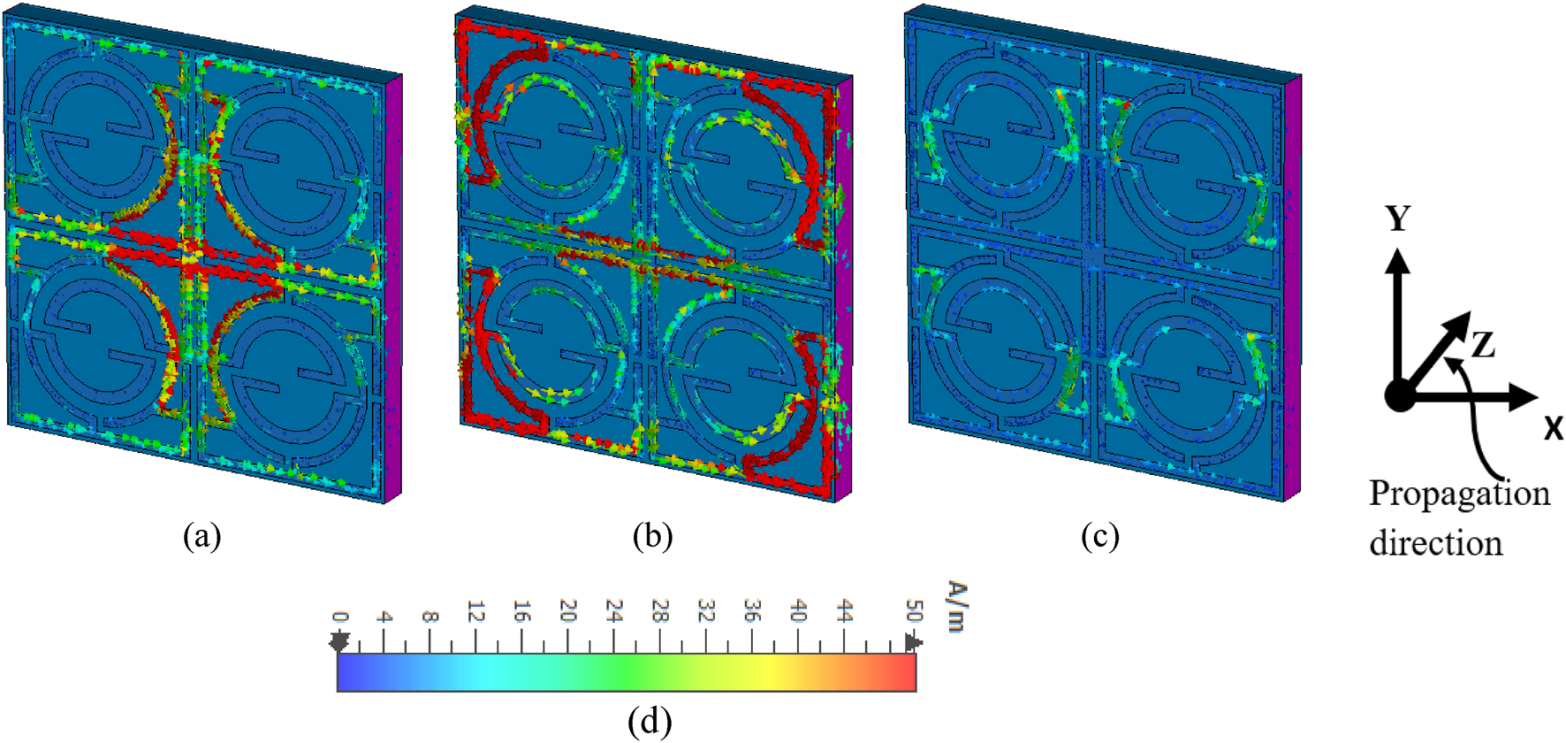
Surface current distribution on MTM: (**a**) 1.98 GHz, (**b**) 4.74 GHz, (**c**) 8.34 GHz, (**d**) scale with color coded bar for the magnitude of the current **(**CST microwave studio suite 2019).

**Figure 11:**
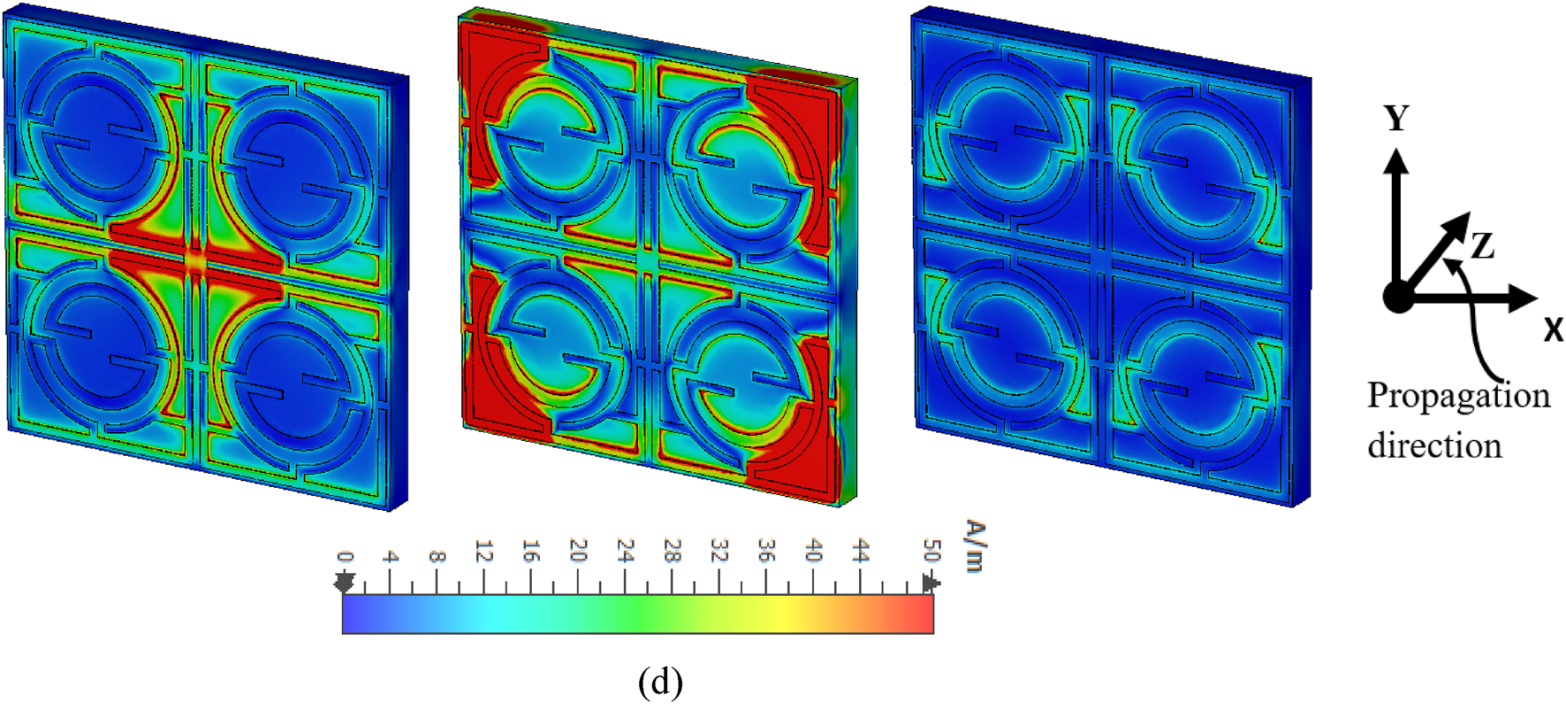
Magnetic (H) field distribution on MTM: (**a**) 1.98 GHz, (**b**) 4.74 GHz, (**c**) 8.34 GHz, (**d**) scale with color coded bar for the magnitude of H field **(**CST microwave studio suite 2019).

**Figure 12:**
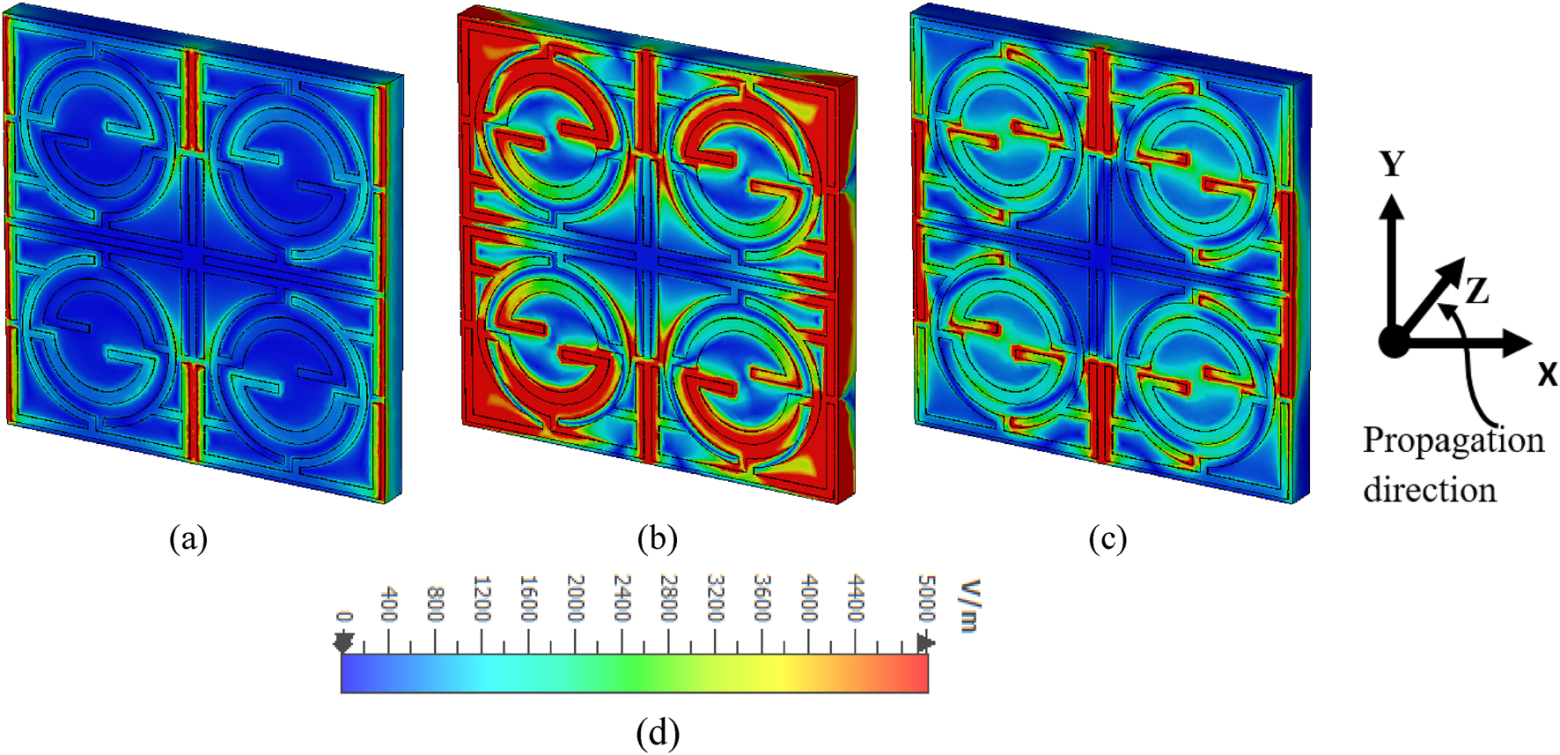
Electric (E) field distribution on MTM: (**a**) 1.98 GHz, (**b**) 4.74 GHz, (**c**) 8.34 GHz, (**d**) scale with color coded bar for the magnitude of the E field (CST microwave studio suite 2019).

The responses of the MTM for different resonances are further analyzed by studying current and various field (magnetic and electric) distributions on the MTM at different frequencies of resonance. Figures 10, 11 and 12 show the surface current, electric, and magnetic field distribution respectively for three different resonance frequencies at 1.98 GHz, 4.74 GHz, and 8.38 GHz. The interrelation between these currents and fields can be made using Maxwell’s curl equations^38^, whereas material interactions with current and field can be described by the equations $\mathrm{D}=\mathrm{\varepsilon E}$ and $\mathrm{B}=\mathrm{\mu H}$. An investigation of Fig. 10a shows that a strong circulating current flows through the central portion of the MTM unit cell. Although the current in the middle ring of each quartile is flowing in the opposite direction, two main current loops are formed at the upper- and lower-middle portions of the cell that eventually create strong magnetic dipoles between the upper- and lower halves. This strong magnetic field (shown in Fig. 11a) contributes to the resonances at 1.98 GHz. The corresponding electric field distribution is shown in Fig. 12a and is more concentrated at the two vertical sides, which also create electric dipoles. Thus, these localized electric and magnetic dipoles help resonances at 1.98 GHz. Now, at 4.74 GHz, more-concentrated currents are noticed at the four corners of the unit cell as depicted in Fig. 10b. The current at the mid-horizontal portion reduces significantly compared to the current at 1.98 GHz. Current that flows at the four corners creates four localized current loops, and the direction of the current at each loop is opposite to the other. Thus, this oppositely directed current at each quartile creates a localized magnetic field (shown in Fig. 11b), but the dipole moments are not as strong compared to the same at 1.98 GHz. By contrast, a strong electric field observed at 4.74 GHz is more evenly distributed over all the cells except for the central region, where the field strength is nearly zero (shown in Fig. 12b). At 8.34 GHz, the current through the resonator decreases significantly, and a moderate amount of current flows through the junction regions of three rings of each quartile, as depicted in Fig. 10c. The associated magnetic fields due to these currents are localized to the vicinity of the current surface (shown in Fig. 11c) and are not as strong and do not contribute to strong dipole formation. Contrary to this, the electric field distribution is more concentrated and localized, as shown in Fig. 12c. The electric field is more directional on the X-axis, and strong electrical dipoles are observed that contribute dominantly to the formation of resonance at 8.34 GHz. Thus, different segments of the unit cell contribute to the resonances at different frequencies by interacting with the incident electromagnetic waves.

### Measured results and discussions

**Figure 13:**
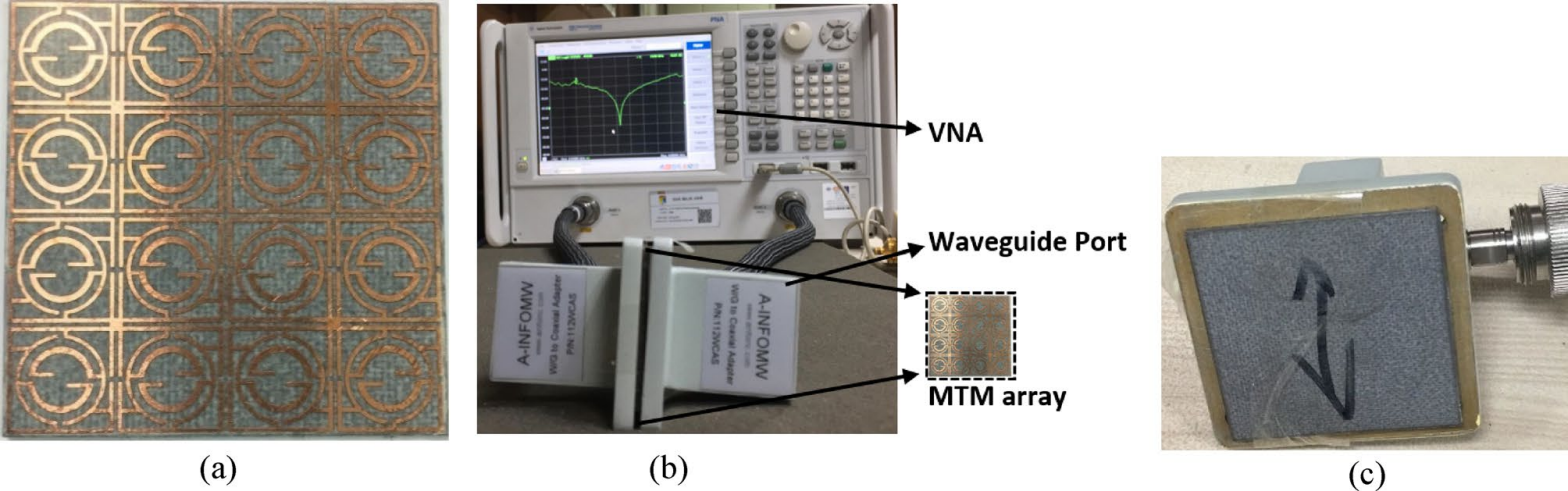
(**a**) Fabricated prototype of 2 × 2 array of proposed MTM unit cells. (**b**) Experimental setup to determine S_21_ using VNA and waveguide port, (**c**) placement of MTM array with the waveguide adapter.

**Figure 14:**
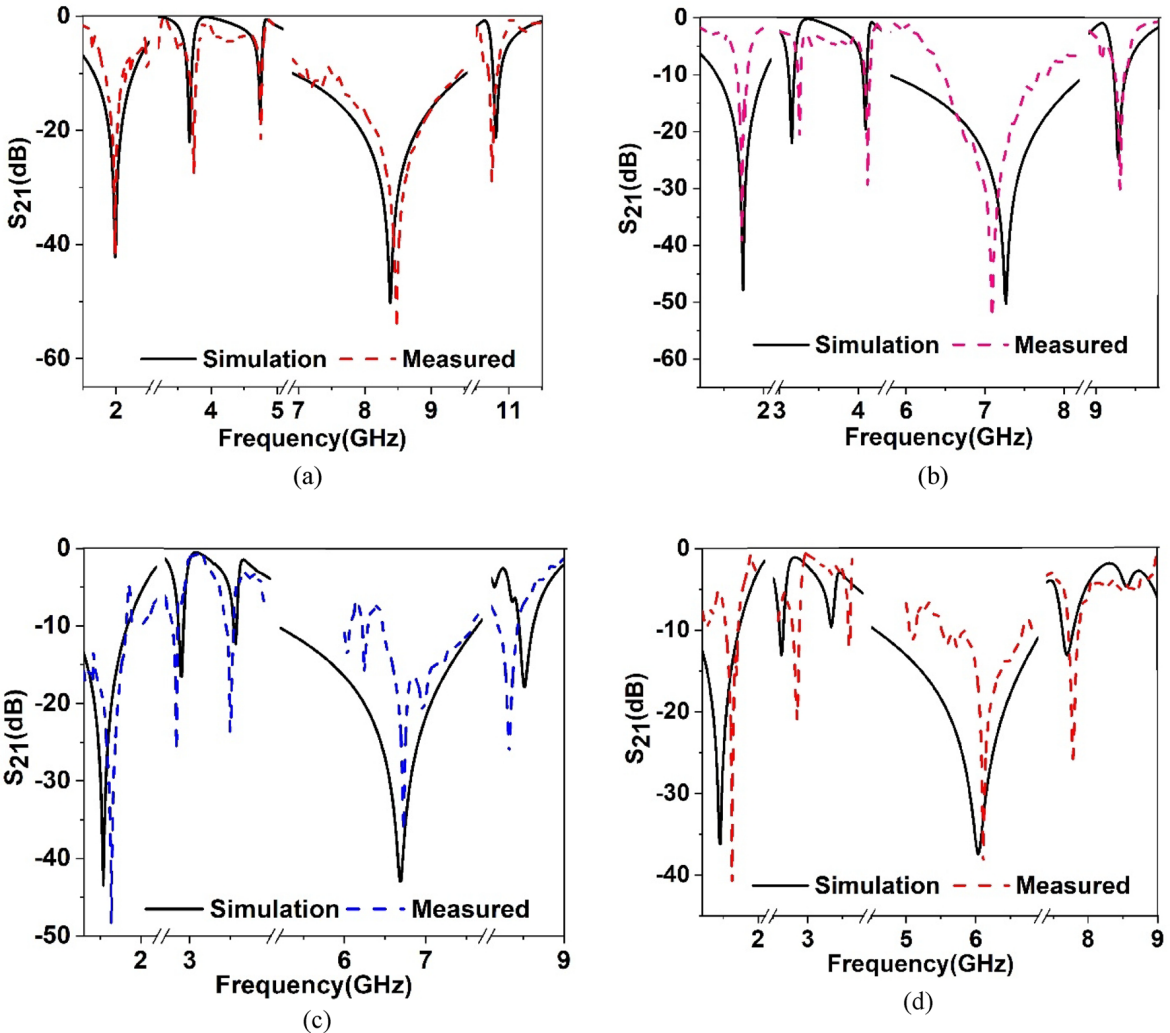
Measured transmission coefficient(S_21_) of the proposed MTM without and with dielectric layer: (**a**) MTM without dielectric layer, (**b**) with Rogers(RT5880) layer, (**c**) with polycarbonate layer, (**d**) with FR4 layer.

The verification of the simulated result is performed through prototype development and performance measurement. The MTM exhibits the same response in the case of an array and unit cell due to the symmetry in design, which is verified through simulation, but for brevity, the simulated result is not included. An MTM unit cell, 2 × 2 array, and 4 × 7 arrays of unit cells are utilized for the measurement purpose since different sets of waveguide adapters are utilized for this measurement as each adapter operates for a particular range of frequency. Such as the waveguide adapter used in the frequency range 1.4–2.2 GHz has a rectangular gap dimension of 65 mm × 130 mm, whereas the adapter used in the frequency range 7 GHz to 10 GHz has the dimensions of 12.5 mm × 28.5 mm. The other dimensions are 43 mm × 86 mm, 22.5 mm × 47 mm, 16 mm × 35 mm, and 9.5 mm × 19 mm respectively for the frequency ranges 2.2–3.3 GHz, 3.95–5.85 GHz, 5.85–8.2 GHz, and 10–15 GHz, respectively. Since the dimension varies for the different frequencies, two sets of arrays, one bears the dimension of 40 mm × 40 mm and another one is 80 mm × 140 mm, as well as one MTM cell of 20 mm × 20 mm are utilized for measuring the performance. Figure 13a illustrates the fabricated prototype of an array of the proposed unit cell, whereas the measurement setup is presented in Fig. 13b. In the measurement process, a vector network analyzer (VNA) is used that is connected to two waveguide ports through the cables, as shown in Fig. 13b. The two ports act as sending and receiving ports, and the MTM device is placed between these two ports. The placement of the MTM array on the waveguide face is shown in Fig. 13c in which the resonator side of the MTM device is positioned to the face of the port 1 waveguide adapter using thin nonmetallic tape. Since the dimensions of the metamaterial array are not exactly equal to the rectangular gap of a waveguide, in some cases portion of the MTM array falls on the metallic body of the waveguide but a thin layer of plastic foil attached to the waveguide face helps to isolate the metallic part of waveguide adapter from MTM metallic part. In the case of multilayer measurement, this isolation is further achieved through a dielectric layer over the resonator. The measured result is illustrated in Fig. 14a for the MTM only. A comparison with the transmission coefficient result obtained in simulation with the measured one reveals that the measured result exhibits a similar response covering the L, S, C, and X bands. Additional local resonance of lower amplitude is observed near 7 GHz in the measured result in comparison with the simulation result. A small deviation of resonance frequencies is noticed for the resonances around 3.67 GHz, 8.38 GHz, and 10.8 GHz with a percentage deviation of 2%, 1.2%, and 0.5%, respectively. The error in the fabrication process, the coupling effect between the two waveguide ports, calibration errors, and a small mismatch in placing the sample between the waveguide ports may be reasons for the mismatching and harmonics. However, disregarding this minor disagreement, the measured result replicates the simulated results well. Furthermore, the frequency-tuning effect is experimented with by using different dielectric layers over the MTM resonator, namely Rogers (RT5880) of 1.57 mm thickness, FR4 of 1.6 mm thickness, and polycarbonate of 1.5 mm thickness. Figure 14b shows the impact of Rogers (RT5880) on the MTM that exhibits resonances at 1.74 GHz, 3.25 GHz, 4.17 GHz, 7.08 GHz, and 9.3 GHz with a small deviation compared to the simulated result. Similarly, the effect of polycarbonate material on the resonator is examined and the measured result is depicted in Fig. 14c in comparison with the simulated result. The measured S_21_ exhibits resonances at 1.64 GHz, 2.85 GHz, 3.48 GHz, 6.73 GHz, and 8.33 GHz. By contrast, as presented in Fig. 14d, the measured result for the FR4 dielectric layer exhibits resonances at 1.6 GHz, 2.8 GHz, 3.6 GHz, 6.11 GHz, and 7.78 GHz, thus indicating larger shifts compared to the initial MTM resonances. Noteworthy to mention that additional local resonances are also noticed within 6–6.5 GHz and within 7–7.5 GHz along with the peak resonance at 6.73 GHz in the case of MTM with polycarbonate. On the other hand, MTM with FR4 dielectric layer exhibits such resonance around 5 GHz. Although a mismatch is obtained between the measured and the simulation results for different dielectric layers, these layers provide frequency-tuning flexibility, and by using different dielectric materials, it is possible to control the resonances according to a particular application.

### Comparative study

A comparison is made between the performance of the proposed MTM structure with several recent works on the MTM based on the dimensions, resonance frequencies, bandwidth, and covering bands; this is displayed in Table. Moreover, the effective medium ratio (EMR) is considered as another comparison parameter that indicates the compactness of the MTM, comparing its dimension to the wavelength using the relation, $EMR=\frac{\lambda }{L}$ , with wavelength, λ  calculated at the lowest frequency of resonance and L being the highest dimension of the structure. The MTMs presented in references^26,39^ exhibit high EMR values compared to our proposed MTM. However, a major constraint of the MTM of reference^26^ is that it operates at a high frequency, and its performance at high frequency will degrade due to the FR4 substrate used since this substrate material suffers more losses at high frequencies. By contrast, the MTM in reference^39^ exhibits only two resonances in the C band. Additionally, those in references^30,40^ exhibit polarization-independent properties but lag behind our presented work in comparison to the number of covering bands and EMR. The rest of the MTMs presented in Table 4 show lower EMR values, cover fewer frequency bands, and also have a lower number of resonances compared to our proposed MTM. Moreover, our proposed structure exhibits polarization-insensitive characteristics that are absent in most of the MTMs presented in Table 4. In addition, the proposed MTM resonance frequency can be controlled using a dielectric layer above the resonator, which adds an extra feature to its performance and makes it suitable for frequency-selective applications.

**Table 4:** Comparison of the performance of the proposed MTM with some other existing works.

Reference	Year	Physical, Electrical dimension (mm × mm, λ × λ)	Resonance frequency (GHz)	Covering bands	EMR
^25^	2017	5 × 5 0.23λ × 0.23λ	13.9, 27.5	Ku, K	4.4
^26^	2020	9 × 9 0.124λ × 0.124λ	14.93, 10.84, 4.15	C, X, Ku	8.03
^27^	2017	25 × 25 0.23λ × 0.23λ	2.8	S	4.28
^28^	2021	10 × 10 0.14λ × 0.14λ	4.2, 10.14, 13.15, 17.1	C, X, Ku	7.17
^29^	2020	10.16 × 22.86 0.32λ × 0.72λ	9.4	X	1.4
^30^	2021	8 × 8 0.23 λ × 0.23 λ	8.67, 13.84	X, Ku	4.3
^39^	2018	6 × 4 0.1 λ × 0.07 λ	5.4, 6.2	C	9.3
^41^	2019	20 × 20 0.16 λ × 0.16 λ	2.4, 3.9, 5.2	S, C	6.25
^42^	2020	6 × 5 0.8λ × 0.67λ	40	Ka	1.25
^40^	2018	20 × 20 0.77λ × 0.77λ	11.5, 13.5	X, Ku	1.4
Proposed	2021	20 × 20 0.13 λ × 0.13 λ	1.98, 3.67, 4.74, 8.38, 10.8	L, S, C, X	7.57

## Conclusion

The metamaterial presented in this article exhibits resonances of S_21_ that occurred at 1.98 GHz, 3.67 GHz, 4.74 GHz, 8.38 GHz, and 10.8 GHz covering the L, S, C, and X bands. The resonator patch is fabricated on a low-loss Rogers (RT5880) substrate of 0.13λ × 0.13λ × 0.01λ electrical dimensions, and the unit cell can be divided into four equal quartiles with each one containing three interconnected modified split rings. The uniqueness in design is achieved by placing the quartiles in a mirror-symmetric pattern so that the coupling effect of one portion is neutralized by the counter-field of the other portion. Thus, this novel structure helps to eliminate the mutual coupling effect in an array of unit cells. Electromagnetic characterization is performed with simulation in CST microwave studio. The obtained result in CST exhibits a negative permittivity property with near-zero permeability and refractive index in the vicinity of the resonances with small power dissipations. The proposed MTM expresses a good EMR value of 7.57, indicating that dimension is much less compared with the wavelength. Furthermore, the resonance frequency of the proposed MTM can be tuned using a dielectric-based multilayer approach. The simulation result of the proposed MTM is verified through measurement, and the simulated and measured S_21_ exhibits good similarity with a minimal amount of deviation. Moreover, frequency tuning is also experimented with using dielectric materials of the same dimension of the MTM, namely Rogers (RT5880), FR4, and polycarbonate having a thickness of 1.57 mm, 1.6 mm, and 1.5 mm, respectively, and the measured result shows good frequency shifting. Thus, this experiment validates that the frequency of the proposed MTM can be tuned according to the required frequency for particular applications. The frequency shifting is more pronounced in the X band, indicating that the proposed MTM can be used for material-sensing applications as well as frequency-selective applications. Furthermore, due to its compact dimension, negative permittivity, near-zero permeability, and refractive index, this metamaterial can be applied for performance enhancement of microwave devices, especially for quality enhancement of microwave antennas.
